# Novel prognostic nomograms in cervical cancer based on analysis of 1075 patients

**DOI:** 10.1002/cam4.5335

**Published:** 2022-11-16

**Authors:** Qunxian Rao, Xue Han, Yuan Wei, Hui Zhou, Yajie Gong, Meimei Guan, Xiaoyan Feng, Huaiwu Lu, Qingsong Chen

**Affiliations:** ^1^ Department of Gynaecological Oncology Sun Yat‐sen Memorial Hospital, Sun Yat‐sen University Guangzhou China; ^2^ Guangdong Provincial Engineering Research Center of Public Health Detection and Assessment Guangdong Pharmaceutical University Guangzhou China; ^3^ School of Public Health Guangdong Pharmaceutical University Guangzhou China

**Keywords:** cervical cancer, disease‐free survival, nomogram, overall survival, prognostic factor

## Abstract

**Objective:**

To explore the factors affecting the prognosis of cervical cancer (CC), and to construct and evaluate predictive nomograms to guide individualized clinical treatment.

**Methods:**

The clinicopathological and follow‐up data of CC patients from June 2013 to December 2019 in Sun Yat‐sen Memorial Hospital of Sun Yat‐sen University were retrospectively analyzed. Log‐rank test was used for univariate survival analysis, and Cox multivariate regression was used to identify independent prognostic factors, based on which nomogram models were established and evaluated in multiple aspects.

**Results:**

Patients were randomly assigned into the training (*n* = 746) and validation sets (*n* = 329). Survival analysis of the training set identified cervical myometrial invasion, parametrial involvement, and malignant tumor history as prognosticators of postoperative DFS and pathological type, cervical myometrial invasion, and history of STD for OS. C‐index was 0.799 and 0.839 for the nomograms for DFS and OS, respectively. Calibration curves and Brier scores also indicated high performance. Importantly, decision curve analysis suggested great clinical applicability of these nomograms.

**Conclusions:**

In this study, we analyzed a cohort of 1075 CC patients and identified DFS‐ or OS‐associated clinicohistologic characteristics. Two nomograms were subsequently constructed for DFS and OS prognostication, respectively, and showed high performance in terms of discrimination, calibration, and clinical applicability. These models may facilitate individualized treatment and patient selection for clinical trials. Future investigations with larger cohorts and prospective designs are warranted for validating these prognostic models.

## Graphical abstract

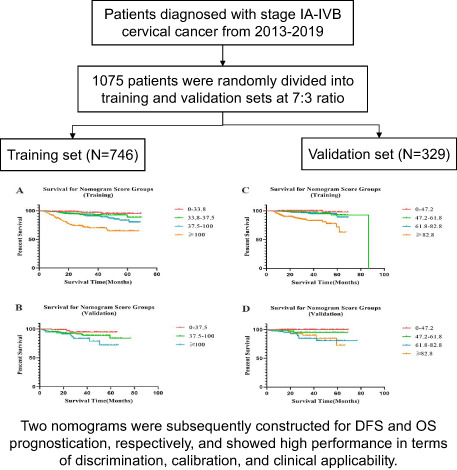

## 1 INTRODUCTION

Cervical cancer (CC) is one of the most common gynecological malignancies. In 2020, there were approximately 604,000 CC cases and 342,000 related deaths worldwide.^1^ In a study involving 184 countries, CC was the most common cancer diagnosis in 45 countries.^2^ The incidence of CC is rising year by year,^3^ and a Latin American study showed a tendency of increasing proportion of younger patients.^4^ Nearly all cases of CC can be attributed to infection with human papillomavirus (HPV). However, a Chinese study suggested a growing patient population despite HPV vaccination programs.^5^ Moreover, although most patients can be cured by local treatment, approximately 20% patients develop recurrence and/or metastasis.^6^ Therefore, CC is likely to impose a heavier burden on public health and cancer care.

A handful of independent prognostic factors have been proposed to date, including tumor size, vascular invasion, and pelvic lymph node (PLN) metastasis, although consensus has not been established for all.^7^, ^8^, ^9^, ^10^, ^11^, ^12^ A study on 110 patients with early stage CC who received surgical intervention showed that PLN metastasis affected prognosis.^8^ In particular, Zheng et al.^13^ proposed a nomogram model for stage IA1‐IIA patients, which identified PLN metastasis as a strong poor prognosticator.

In this retrospective study, we identified clinicopathological factors associated with overall and DFS of 1075 Chinese CC patients, based on which a nomogram model was subsequently constructed and validated. In addition, we also measure the clinical applicability of the model using decision curve analysis.

## 2 PATIENTS AND METHODS

### 2.1 Patients

**FIGURE 1:**
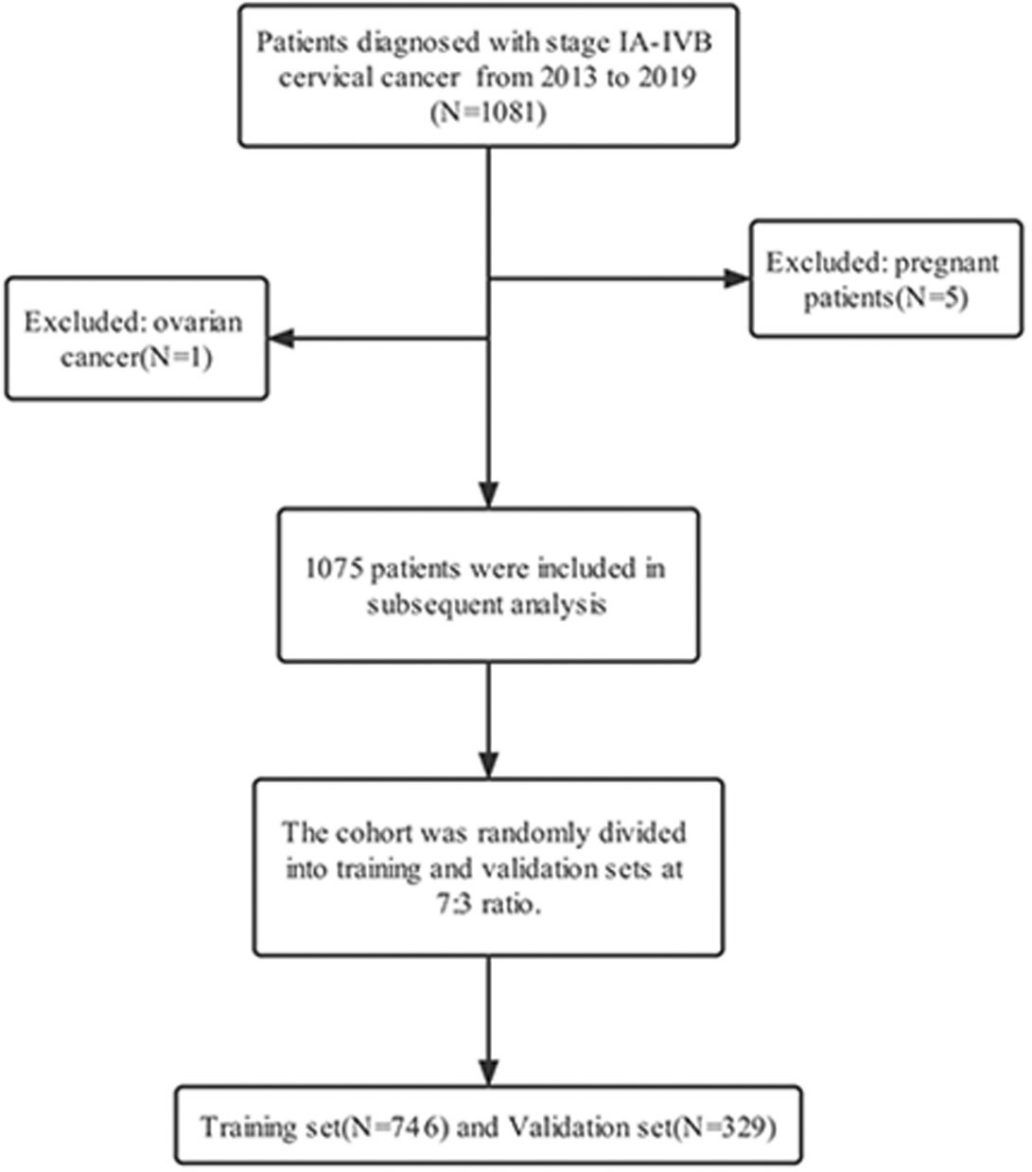
Study selection process.

We included 1081 women with stage IA‐IVB CC who received treatment at the Sun Yat‐sen Memorial Hospital of Sun Yat‐sen University in China from June 2013 to December 2019. After excluding pregnant patients (*n* = 5) or those who also had ovarian cancer (*n* = 1), data for 1075 cases were used for subsequent analyses (Figure 1).

Information regarding clinicopathological features and follow‐up were retrieved from the electronic medical records. Interrogated clinicopathological features included age at diagnosis, FIGO stage, menopause, ovarian metastasis (resection), parity, history of STD, history of malignant tumor, diabetes, liver disease, cardiovascular and cerebrovascular diseases, histological type, tumor differentiation, PLN metastasis status, para‐aortic lymph node metastasis status, tumor size, vascular invasion, cervical junction invasion, cervical myometrial invasion, parametrial invasion, vaginal fornix involvement. Follow‐up data were collected regarding occurrence of and time to disease recurrence, metastasis, and death. DFS was defined as time interval from surgical excision to disease recurrence or death, and OS was defined as the interval from diagnosis to death or last follow‐up. The study was conducted in accordance with the ethical standards laid down in the Declaration of Helsinki. All participants have signed informed consent, and institutional Review Board approval was obtained from the Medical Ethics Board at the Sun Yat‐sen Memorial Hospital.

### 2.2 Statistical analysis

The cohort was randomly divided into training and validation sets at 7:3 ratio. The training set was subjected to Kaplan–Meier and log‐rank analyses to examine their association with DFS or OS, and factors with *p*‐values of <0.1 were selected for multivariate Cox regression analysis. The results of Cox regression were then used to establish prognostic nomograms.

Nomograms were evaluated for discrimination ability with the consistency index (C‐index) and area under curve (AUC) of the corresponding receiver operating characteristic (ROC).^14^ Calibration curves were plotted to verify the accuracy and reliability of the nomogram. The Brier score was also used to evaluate the overall performance of the model.^15^ Decision curve analysis (DCA) was also performed to show the clinical usefulness of the nomogram.

All statistical analyses were performed with SPSS (25.0), GraphPad Prism (8.4.3) and the R programming language (4.0.5). The R statistical packages “rms,” “survival,” “foreign,” and “survivalROC” were used to calculate the C‐index and plot the calibration curves, the ROC curves. All statistical tests were two‐sided with the significance level set at 0.05.

## 3 RESULTS

### 3.1 Demographic and clinical characteristics

The 1075 patients included in this study were randomly assigned into training (*n* = 746) or validation set (*n* = 329). Age at CC diagnosis age was 22–79 (median 49) years in the intact cohort, 22–79 (median 49 years) in the training set, and 22–79 (median 48 years) in the validation set. The two sets also had similar durations of follow‐up, with a median of 34.38 (range 0.20–86.80) months for the training set and 31.03 (0.00–77.40) months for the validation set. Other important clinicopathologic features were also comparable between the two sets, including histologic subtype, metastasis to the ovaries, tumor size, rate of vascular, cervical junction, or myometrial invasion, vaginal fornix involvement, history of malignancies or sexually transmitted disease (STD), and other medical conditions (Table 1). In particular, the PLN metastases ratio were 23.7% and 18.2%, and the para‐aortic lymph node metastasis ratio were 0.8% and 0.9% in training and validation sets, respectively.

**TABLE 1:** Demographic and clinicopathologic characteristics of the 1075 included patients

Feature	Total (*n* = 1075)	Training set (*n* = 746)	Validation set (*n* = 329)
Age			
<30	43 (4.0%)	30 (4.0%)	13 (4.0%)
30–60	887 (82.5%)	611 (81.9%)	276 (83.9%)
≥60	145 (13.4%)	105 (14.1%)	40 (12.1%)
FIGO stage			
I	816 (75.9%)	558 (74.8%)	258 (78.4%)
II	222 (20.7%)	162 (21.7%)	60 (18.2%)
III‐IV	37 (3.4%)	26 (3.5%)	11 (3.4%)
Pathological type			
SCC	823 (76.6%)	582 (78.0%)	241 (73.3%)
AC	166 (15.4%)	105 (14.1)	61 (18.5%)
Other	86 (8.0%)	59 (7.9%)	27 (8.2%)
Vascular invasion			
No	619 (57.6%)	408 (54.7%)	211 (64.1%)
Yes	456 (42.4%)	338 (45.3%)	118 (35.9%)
Cervical junction invasion			
No	747 (69.5%)	522 (70.0%)	225 (68.4%)
Yes	328 (30.5%)	224 (30.0%)	104 (31.6%)
Myometrial invasion			
No	230 (21.4%)	159 (21.3%)	71 (21.6%)
<2/3	355 (33.0%)	239 (32.0%)	116 (35.3%)
2/3‐all	302 (28.1%)	213 (28.6%)	89 (27.0%)
All	188 (17.5%)	135 (18.1%)	53 (16.1%)
Parametrial involvement			
No	1058 (98.4%)	734 (98.4%)	324 (98.5%)
Yes	17(1.6%)	12 (1.6%)	5 (1.5%)
Vaginal fornix involvement			
No	852 (79.3%)	583 (78.2%)	269 (81.8%)
Yes	223 (20.7%)	163 (21.8%)	60 (18.2%)
Ovarian metastasis			
No	1064 (99.0%)	738 (98.9%)	326 (99.1%)
Yes	11 (1.0%)	8 (1.1%)	3 (0.9%)
History of malignant tumor			
No	1059 (98.5%)	735 (98.5%)	324 (98.5%)
Yes	16 (1.5%)	11 (1.5%)	5 (1.5%)
Tumor diameter			
<4 cm	856 (79.6%)	593 (79.4%)	263 (79.9%)
4‐8 cm	211 (19.6%)	146 (19.6%)	65 (19.8%)
≥8 cm	8 (0.7%)	7 (1.0%)	1 (0.3%)
History of STD			
No	1047 (97.4%)	724 (97.1%)	323 (98.2%)
Yes	28 (2.6%)	22 (2.9%)	6 (1.8%)
Diabetes			
No	980 (91.2%)	676 (90.6%)	304 (92.4%)
Yes	95 (8.8%)	70 (9.4%)	25 (7.6%)
With cardiovascular and cerebrovascular diseases			
No	906 (84.3%)	625 (83.8%)	281 (85.4%)
Yes	169 (15.7%)	121 (16.2%)	48 (14.6%)
Pelvic lymph node metastasis			
No	838 (78.0%)	569 (76.3%)	269 (81.8%)
Yes	237 (22.0%)	177 (23.7%)	60 (18.2%)
Para‐aortic lymph node metastasis			
No	1066 (99.2%)	740 (99.2%)	326 (99.1%)
Yes	9 (0.8%)	6 (0.8%)	3 (0.9%)

### 3.2 Prognostic factors predicting postoperative survival of CC patients

We started identifying prognostic factors for postoperative survival by interrogating association between each factor and DFS at *p*‐values with. Univariate log‐rank analysis revealed a lack of prognostic significant for factors such as age and presence of para‐aortic lymph node metastasis (Table 2). In contrast, presence of PLN metastasis was strongly associated with DFS. There was a trend toward association between postoperative survival and history of STD or concurrent cardiovascular and cerebrovascular diseases, while a few other factors showed significant association with DFS, including tumor diameter, cervical junction invasion, myometrial invasion, parametrial involvement, vaginal fornix involvement, and history of malignant tumor. Multivariate Cox regression analysis was then performed with factors that *p* < 0.1. The results indicated that cervical myometrial invasion, parametrial involvement, and history of malignant tumor were independent prognostic factors for DFS in CC (Table 3).

**TABLE 2:** Log‐rank analysis of prognostic factors for DFS and OS

Feature	DFS		OS	
	χ2	*p‐value*	χ2	*p‐value*
Age	3.329	0.189	2.474	0.290
FIGO stage	12.595	0.002	10.173	0.006
Pathological type	6.857	0.032	9.987	0.007
Tumor diameter	20.962	<0.001	20.198	<0.001
Vascular invasion	11.723	0.001	14.921	<0.001
Cervical junction invasion	14.433	<0.001	21.065	<0.001
Myometrial invasion	58.720	<0.001	53.969	<0.001
Parametrial involvement	26.027	<0.001	11.116	0.001
Vaginal fornix involvement	20.172	<0.001	8.044	0.005
Ovarian metastasis	8.428	0.004	7.338	0.007
History of malignant tumor	18.472	<0.001	0.617	0.432
History of STD	4.099	0.043	7.430	0.006
With cardiovascular and cerebrovascular diseases	3.842	0.050	0.012	0.912
Diabetes	10.131	0.001	6.683	0.010
Pelvic lymph node metastasis	13.256	<0.001	18.015	<0.001
Para‐aortic lymph node metastasis	0.416	0.519	0.288	0.592

**TABLE 3:** Multivariate Cox regression analysis of OS/DFS prognostic factors

Feature	DFS		OS	
	*p‐value*	HR (95% CI)	*p‐value*	HR (95% CI)
Myometrial invasion	<0.001		<0.001	
No	—	1.000 (reference)	—	1.000 (reference)
<2/3	0.085	2.124 (0.901–5.010)	0.107	5.442 (0.694–42.677)
2/3‐all	0.131	1.972 (0.817–4.758)	0.033	9.188 (1.194–70.715)
All	<0.001	7.443 (3.276–16.915)	<0.001	36.143 (4.885–267.395)
Parametrial involvement	0.023	2.742 (1.148–6.553)	NI	
History of malignant tumor	<0.001	6.810 (2.729–16.992)	NI	
Pathological type	NI		0.002	
SCC			—	1.000 (reference)
AC			0.149	1.781 (0.813–3.904)
Other			0.001	4.136 (1.859–9.201)
History of STD	NI		0.016	3.587 (1.272–10.121)

Abbreviation: NI, not included in analysis.

The same approach was applied to identify OS‐associated clinicohistologic features, which included FIGO stage, pathological type, vascular invasion, cervical junction invasion, myometrial invasion, parametrial involvement, vaginal fornix involvement, PLN metastasis, and history of STD per univariate analysis. Multivariate Cox regression analysis showed that cervical myometrial invasion, pathological type, and history of STD were independent prognostic factors for postoperative OS (Table 3).

### 3.3 Construction and evaluation of nomograms for DFS or OS prediction

**FIGURE 2:**
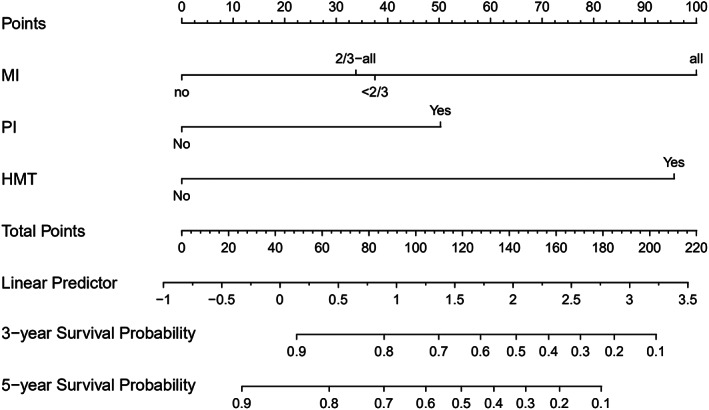
A nomogram for predicting DFS. HMT, history of malignant tumor; MI, myometrial invasion; PI, parametrial involvement

**FIGURE 3:**
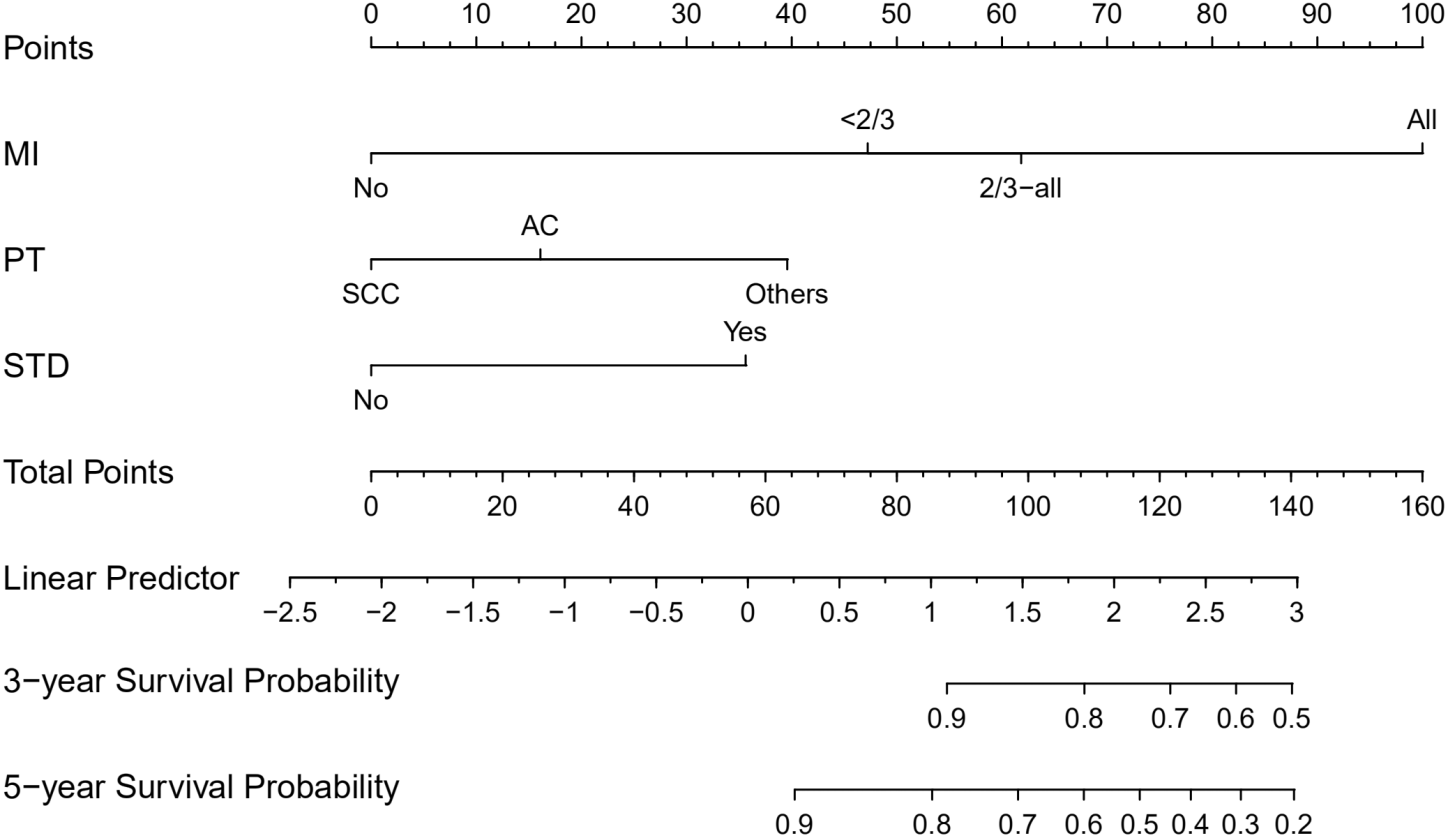
A nomogram for predicting postoperative OS. MI, myometrial invasion; PT, pathological type; STD, sexually transmitted diseases

Cervical myometrial invasion, parametrial involvement, and history of malignant tumor were used to construct a nomogram model for predicting DFS and 3‐ and 5‐year DFS rates (Figure 2), and pathological type, cervical myometrial invasion, and history of STD were used to construct a nomogram for predicting postoperative OS and 3‐ and 5‐year OS rates (Figure 3).

**FIGURE 4:**
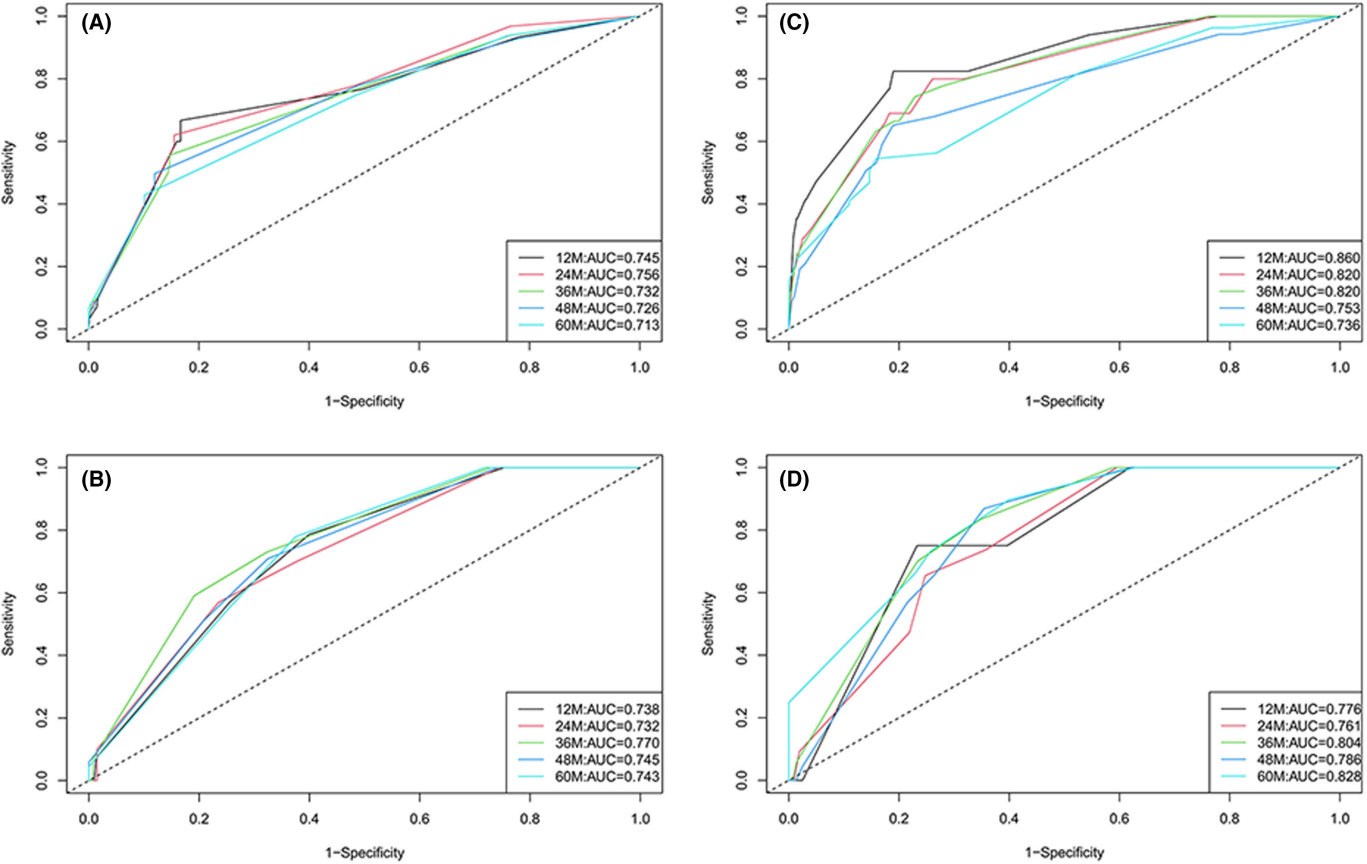
AUC curve. AUCs in 1‐to‐5 year for (A) the DFS nomogram in the training set; (B) the DFS nomogram in the validation set; (C) the OS nomogram in the training set; (D) the OS nomogram in the validation set.

**FIGURE 5:**
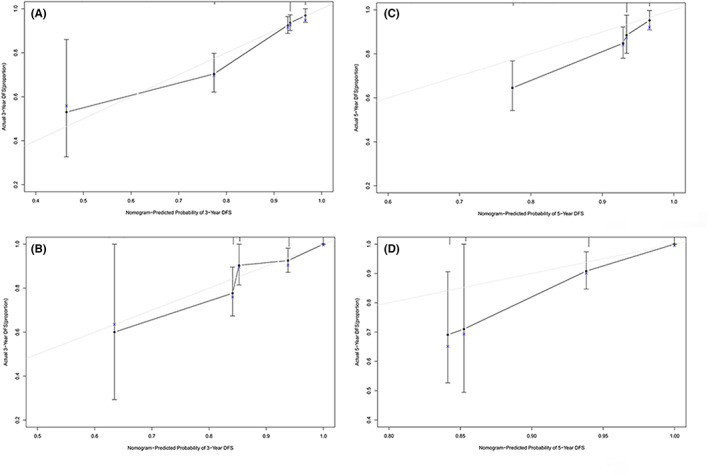
DFS calibration curve. Calibration curves for (A) the 3‐year DFS nomogram in the training set; (B) the 3‐year DFS nomogram in the validation set; (C) the 5‐year DFS nomogram in the training set; (D) the 5‐year DFS nomogram in the validation set.

**FIGURE 6:**
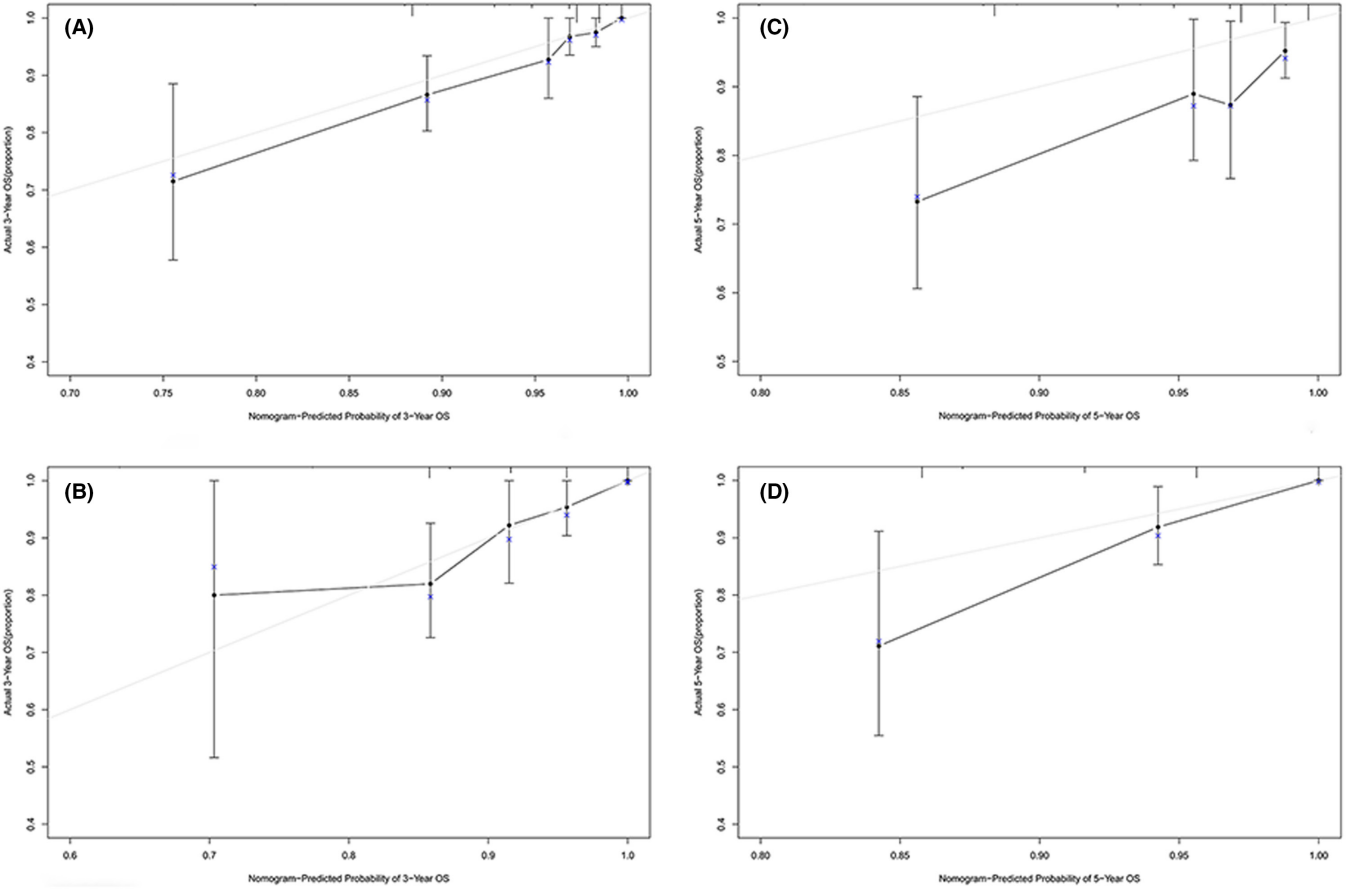
OS calibration curve. Calibration curves for (A) the 3‐year OS nomogram in the training set; (B) the 3‐year OS nomogram in the validation set; (C) the 5‐year OS nomogram in the training set; (D) the 5‐year OS nomogram in the validation set.

**FIGURE 7:**
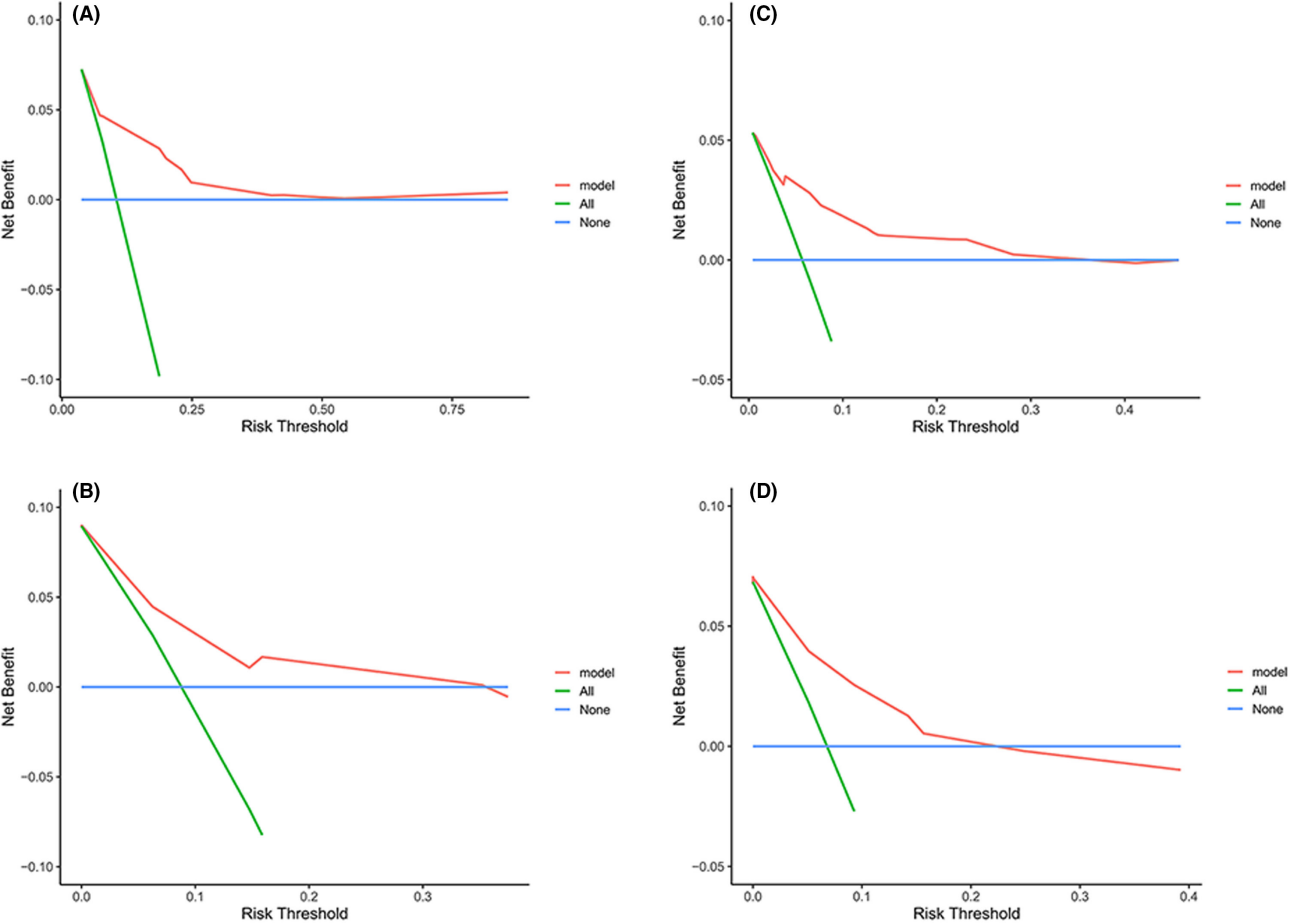
Decision curve. Decision curves for (A) the DFS nomogram in the training set; (B) the DFS nomogram in the validation set; (C) the OS nomogram in the training set; (D) the OS nomogram in the validation set.

We then comprehensively evaluated the nomograms for discrimination, calibration and clinical utility with C‐index, receiver operating characteristic (ROC), Brier score, calibration plot, and DCA analysis (Table 4, Figure 4, Figure 5, Figure 6, Figure 7). The nomogram for DFS showed a C‐index of 0.775 (95% confidence interval: 0.707–0.842) for the training set and 0.799 (0.711–0.888) for validation set. AUCs for 1‐ to 5‐year predictions ranged 0.713–0.756 in the training set and 0.732–0.770 in the validation set (Figure 4). Brier scores for 1‐ to 5‐year predictions ranged 3.67%–12.05% in the training set and 3.95%–12.52% in the validation set.

**TABLE 4:** Evaluation index of nomograms

		DFS rate (95% CI)					OS rate (95% CI)				
		1‐year	2‐year	3‐year	4‐year	5‐year	1‐year	2‐year	3‐year	4‐year	5‐year
Training set	C‐index	0.775 (95%CI:0.707–0.842)					0.831 (95%CI:0.768–0.894)				
	AUC	0.745	0.756	0.732	0.726	0.713	0.860	0.820	0.820	0.753	0.736
	Brier score	3.672%	6.913%	8.759%	10.765%	12.048%	2.004%	3.749%	5.184%	7.013%	9.611%
Validation set	C‐index	0.799 (95%CI:0.711–0.888)					0.839 (95%CI:0.756–0.921)				
	AUC	0.738	0.732	0.770	0.745	0.743	0.776	0.761	0.804	0.786	0.828
	Brier score	3.950%	6.181%	8.443%	9.550%	12.524%	1.195%	3.948%	5.957%	7.695%	8.168%

The nomogram for postoperative OS generally showed slightly better performance in these aspects. The C‐index of 0.831 (0.768–0.894) was for the training set and 0.839 (0.756–0.921) for validation set. AUCs for 1‐ to 5‐year predictions ranged 0.736–0.860 in the training set and 0.761–0.828 in the validation set (Figure 4). Moreover, Brier scores for 1‐ to 5‐year predictions ranged 2.00%–9.61% in the training set and 1.20%–8.17% in the validation set.

Calibration plots for 3‐year and 5‐year DFS/OS rates were plotted, comparing nomogram‐predicted with actual outcomes and indicated high quality of the nomogram (Figure 5, Figure 6). Furthermore, DCA analysis showed the value of the two models. The net benefit of prognostic mnomograms was larger than that in the other two scenarios (all screening or nonscreening) in a wide range of threshold probabilities as displayed in Figure 7.

### 3.4 Performance of the nomogram in stratifying risk of patients

**FIGURE 8:**
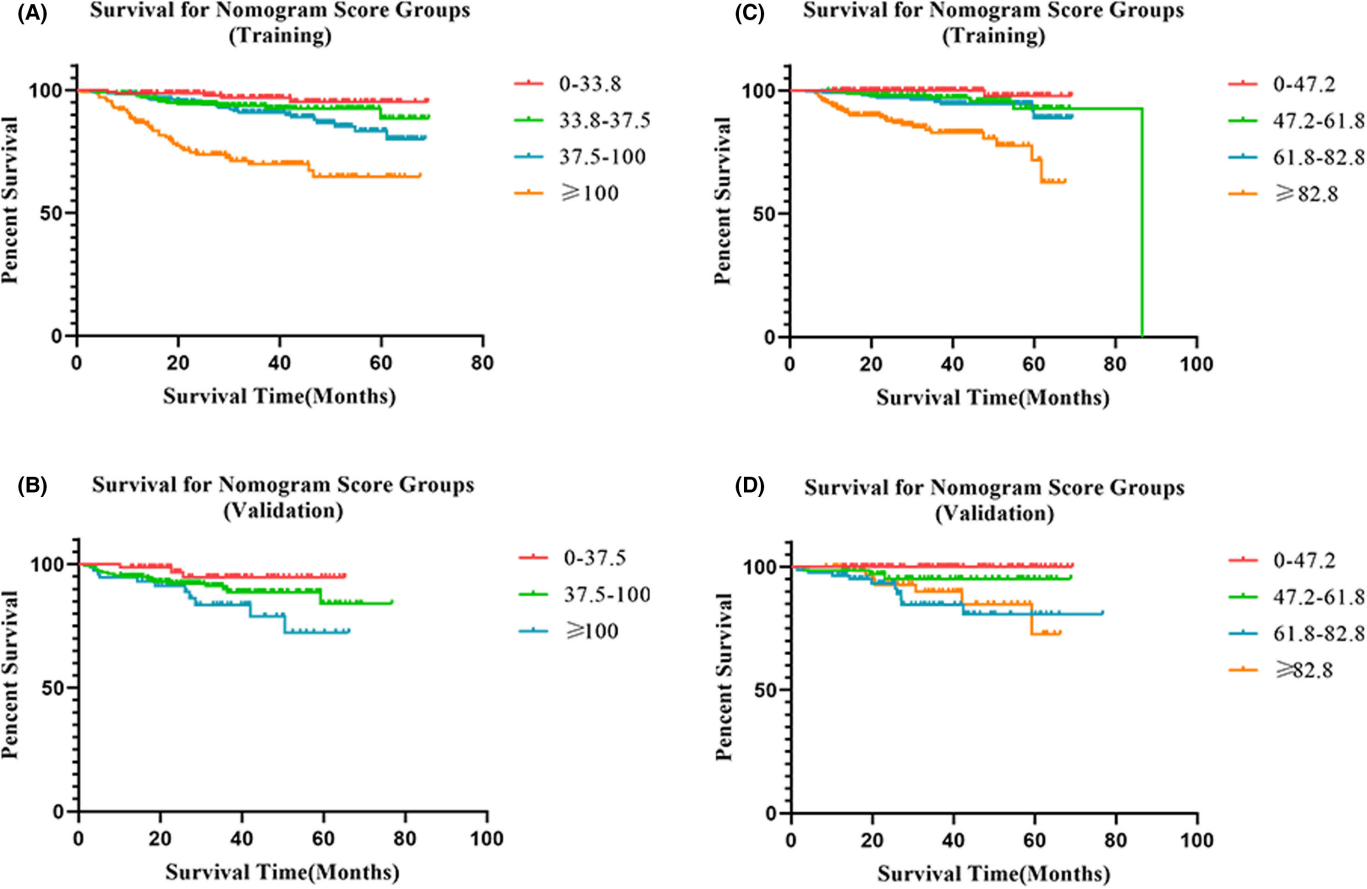
Kaplan–Meier survival curve. Kaplan–Meier survival curves for (A) the DFS nomogram in the training set; (B) the DFS nomogram in the validation set; (C) the OS nomogram in the training set; (D) the OS nomogram in the validation set.

To further analyze the feasibility and validity of the prognostic nomogram, we divided the cervical cancer patients into different subgroups after sorting by total risk score. Survival analyses demonstrated significant distinctions between subgroups in the training set (*p* < 0.05). Same method was performed in the validation set and survival differences were observed among subgroups (*p* < 0.05). As presented in Figure 8, each subgroup established a distinct prognosis, and the corresponding Kaplan–Meier survival curves were delineated respectively.

## 4 DISCUSSION

CC currently ranks the fourth most common cancer according to global statistics.^16^ Identifying clinicopathologic features with prognostic significance and establishing prognosis models may facilitate individualized treatment and patient selection for clinical trials. This study identified cervical myometrial invasion, parametrial involvement and history of malignant tumor as independent prognosticators for DFS and pathological subtype, cervical myometrial invasion and history of STD for OS in CC. Furthermore, a nomogram model was established based on these prognostic factors for predict 3‐ and 5‐ years survival rates and showed good performance in terms of discrimination, calibration, and clinical applicability.

A handful of prognostic factors have been proposed for CC, although consensus remains to be established for some, including pathological factors.^17^, ^18^, ^19^ CC can be classified into squamous cell carcinoma (SCC), adenocarcinoma (AC), adenosquamous carcinoma, clear cell carcinoma, cervical villous tubular papillary adenocarcinoma, and others. The first three subtypes are the most common ones, with SCC accounting for about 85% of CC cases.^20^, ^21^, ^22^ AC is more prone to ovarian metastasis than SCC and is generally considered an important prognostic factor.^23^, ^24^ A study of postoperative disease‐specific survival (DSS) for stage IA2‐IIB cervical cancer also found that AC was associated with poor prognosis.^25^ Our results further suggested AC as independent prognosticator for OS.

In addition to pathological subtype, there is a large body of evidence supporting the prognostic significance of vascular invasion and PLN metastasis in CC.^7^, ^8^, ^9^, ^10^, ^11^ An analysis of 79 CC patients identified vascular invasion, lymph node metastasis and clinical stage as predictors of OS.^12^ In particular, the negative prognostic impact of PLN metastasis was supported by other investigations^9^, ^10^ and was recommended by the National Comprehensive Cancer Network (NCCN) guidelines as a high‐risk factor after surgery.^26^ In our study, vascular invasion and PLN metastasis was identified to have an adverse impact on cervical cancer, but it was not an independent prognostic factor for DFS and OS.

Additionally, a meta‐analysis of 25 studies with a total of 6500 patients suggested prognostic relevance for tumor size in addition to lymph node metastasis and vascular invasion.^27^, ^28^, ^29^ Tumor size is also an intermediate risk factor per NCCN guidelines. There is evidence that tumors with diameters >4 cm were associated with greater risk of disease recurrence.^30^ However, we did not observe a similar significant association for either DFS or OS (*p* > 0.05 for both). This discrepancy may be attributable to the limited number of patients with tumors with diameters >4 cm (*n* = 153 and 66 for the training and validation sets, respectively), thereby reducing statistical power.

NCCN guidelines for CC also recommend parametrial involvement as a high risk factor after surgery, which was supported by a study of 110 early CC patients, which showed associated parametrial involvement with poor prognosis after surgical intervention.^8^ Reported incidence rates of parametrial involvement in early CC vary considerably from 0.6% to 32.5%.^31^, ^32^, ^33^, ^34^ In this study, parametrial involvement occurred in 2.3% stage I and II patients and was identified as an independent poor prognosticator for DFS, thereby supporting previous findings.

Prognostic significance of cervical myometrial invasion remains under debate.^12^ There is evidence linking depth of myometrial invasion and 5‐year survival rate. Our study identified myometrial invasion is an independent prognosticator for both OS and DFS. In contrast, FIGO stage was not significantly associated with DFS or OS despite being an established prognostic factor.^35^, ^36^ This distinction may at least be partly due to the absence of discrimination between stage IA and IB in the electronic medical records.

Notably, we also identified a novel potential poor prognosticator of DFS, i.e. malignant tumor history. This may be related to the decline in physical function caused by the patient's previous suffering from other cancer.^14^ In this study, 16 patients with a history of other malignancies mainly had breast cancer, followed by thyroid cancer. A nomogram incorporating malignant tumor history showed consistent power of discrimination (C‐index = 0.775 and 0.799 for training and validation sets, respectively). To our knowledge, our is the first report linking history of malignant tumor with DFS. In the future, further large‐scale, prospective studies are warranted to validate this finding.

Interestingly, a history of STD was negatively associated with OS in this study. Existing research mainly focuses on the history of STD and CC incidence instead of prognosis. Patients with a history of STD in this study mostly had syphilis, followed by condyloma acuminatum. Syphilis infection has been found necessary for the CC pathogenesis following HPV infection and proposed as a predictor of invasive CC.^37^, ^38^ Condyloma acuminatum is also an HPV‐related condition. Low‐risk HPV infections typically cause condyloma acuminatum,^39^ although high‐risk HPV infections have been found in 31% of condyloma acuminata.^40^ This novel finding in this study, which suggests prognostic relevance for syphilis or condyloma acuminata, warrants further clinical validation and may serve as a basis for insights into the underlying biology.

Nomograms are an established tool for estimating cancer prognosis in quantitative terms and has been adopted in CC.^41^ In a pioneering study, Polterauer and colleagues studied 528 CC cases and constructed a nomogram for OS prediction based on age, FIGO stage, PLN metastasis, percentage of invaded lymph nodes, parametrial invasion, and tumor size citation (Table 5). The C‐index is 0.723, and a calibration chart is created based on internal cross‐validation of bootstrap resampling, which has good consistency and accuracy.^41^ In addition, Zheng et al.^13^ constructed a model for OS in early CC using body mass index, blood albumin level, platelets, white blood cell count, tumor differentiation, and PLN status were independent prognostic factors. The model achieved good discrimination (C‐index = 0.74). In study of 1563 cases, Zhou et al.^42^ predicted 5‐year OS based on pathological subtype, lymph node metastasis, lymphatic vascular space invasion, interstitial invasion, parametrial invasion, and tumor diameter and achieved a C‐index of 0.71. Importantly, a nomogram by Feng et al. Used age, race, histology, extension range, tumor size, radiotherapy and surgery prognostic factor for predicting the OS of stage IIIC1 CC achieved good discrimination (C‐index = 0.687) and was externally verified with SEER data sets. The authors also evaluated the model's clinical adaptability with evaluated by DCA.^43^

**TABLE 5:** Performance evaluations of the nomogram model

Study	Endpoint	C‐index (95% CI)	Brier score	Calibration curve	DCA
This study	DFS, OS	0.775 (95%CI:0.707–0.842); 0.831 (95%CI:0.768–0.894)	Yes	Yes	Yes
Polterauer, Grimm et al.	OS	0.723	No	Yes	No
Liu Q et al.	OS, CSS	0.831 (95%CI:0.815–0.847); 0.855 (95%CI:0.839–0.871)	No	Yes	Yes
Zheng RR et al.	OS	0.74 (95% CI:0.68–0.80)	No	Yes	No
Zhou H et al.	OS	0.71 (95% CI:0.65–0.77)	No	Yes	No
Feng et al.	OS, CSS	0.687;0.692	No	Yes	Yes

Our study established a nomogram for predicting DFS based on cervical myometrial invasion, parametrial invasion and malignant tumor history and one for predicting OS based on pathological type, cervical myometrial invasion and history of STD. As far as we know, we are the first study to link the history of malignant tumor and STD with poor prognosis in CC patients and establish nomograms. In addition, both models were evaluated with C‐index, ROC curve, Brier score, and DCA analysis, all of which indicated high performance and clinical applicability. C‐index for the two models were 0.775 (0.707–0.842) for DFS and 0.831 (0.768–0.894) for OS in the training set and 0.799 (0.711–0.888) for DFS and 0.839 (0.756–0.921) for OS in the validation set. Calibration curves of 3‐year and 5‐year were also created based on internal validation of bootstrap resampling and showed remarkable accuracy.

Decision curve analysis (DCA) can calculate the net benefit of a predictive model to measure its clinical utility. DCA was performed to evaluate the clinical applicability of the constructed nomograms when quantifying the net improvement benefits under different threshold probabilities.^44^ After validation, DCA confirmed that our nomograms have better clinical benefits and utility in predicting the survival of patients with CC.

Despite having achieved prognostic accuracy, our study is not devoid of limitations. In addition to the retrospective, single‐center nature, the nomogram models, which did not undergo validation with an external cohort. Secondly, the calibration curve suggested low performance in predicting 5‐year survival rates for OS and DFS chart is poor, which may be owing to the higher proportion of censored events at this time point.

## 5 CONCLUSION

In this study, we analyzed a cohort of 1075 CC patients and identified DFS‐ or OS‐associated clinicohistologic characteristics. Two nomograms were subsequently constructed for DFS and OS prognostication, respectively, and showed high performance in terms of discrimination, calibration, and clinical applicability. Further research is warranted for validating these nomograms with larger cohorts and prospective studies.

## AUTHOR CONTRIBUTIONS

**Qunxian Rao:** Conceptualization (equal); formal analysis (equal); methodology (equal); writing – original draft (equal). **Xue HAN:** Conceptualization (equal); project administration (equal); writing – original draft (equal); writing – review and editing (equal). **Yuan Wei:** Conceptualization (equal); data curation (equal); writing – review and editing (equal). **Hui Zhou:** Conceptualization (equal); data curation (equal); writing – review and editing (equal). **Yajie Gong:** Conceptualization (equal); data curation (equal); writing – original draft (equal). **Mei‐mei Guan:** Conceptualization (equal); data curation (equal); writing – original draft (equal). **Xiaoyan Feng:** Conceptualization (equal); data curation (equal); writing – original draft (equal). **Huaiwu Lu:** Conceptualization (equal); project administration (equal); resources (equal); supervision (equal). **Qingsong Chen:** Conceptualization (equal); formal analysis (equal); methodology (equal); validation (equal); writing – review and editing (equal).

## FUNDING INFORMATION

This research was supported by Medical Science and Technology Foundation of Guangdong Province (grant No.2019GCZX012 [QSC]).

## CONFLICT OF INTEREST

There is no financial support or financial agreement, bonds, or any other conflict of interest involving the authors and companies, which may have an interest in the publication of this paper.

## REVIEW BOARD/COMMITTEE APPROVAL

Institutional Review Board approval was obtained from the Medical Ethics Board at the Sun Yat‐sen Memorial Hospital (No.SYSEC‐KY‐KS‐2021‐336).

## INFORMED CONSENT

All participants have signed informed consent.

## Supporting information


Appendix S1.

## Data Availability

The data that supports the findings of this study are available in the supplementary material of this article.
